# Shelf-Life Prediction and Thermodynamic Properties of No Added Sugar Chocolate Spread Fortified with Multiple Micronutrients

**DOI:** 10.3390/foods11152358

**Published:** 2022-08-06

**Authors:** Roberta Tolve, Fideline Laure Tchuenbou-Magaia, Lucia Sportiello, Federico Bianchi, Iza Radecka, Fabio Favati

**Affiliations:** 1 Department of Biotechnology, University of Verona, Strada Le Grazie 15, 37134 Verona, Italy; 2 Division of Chemical Engineering, School of Engineering, Computing and Mathematical Sciences, University of Wolverhampton, Wolverhampton WV1 1LY, UK; 3 School of Agricultural, Forestry, Food and Environmental Sciences (SAFE), University of Basilicata, Viale Dell’Ateneo Lucano 10, 85100 Potenza, Italy; 4 School of Science, Faculty of Science and Engineering, University of Wolverhampton, Wolverhampton WV1 1LY, UK

**Keywords:** Arrhenius model, fortification, oxidation, spreadable chocolate, shelf-life, vitamin D

## Abstract

The development of fortified healthy pleasant foods, in which saturated fats are replaced with unsaturated ones, poses a challenge for the food industry due to their susceptibility to oxidative rancidity, which decreases product shelf-life, causes the destruction of health-promoting molecules, and forms potentially toxic compounds. A comparative study applying the Arrhenius model was carried out to investigate the oxidative stability and predict the shelf-life of a newly developed no added sugar chocolate spread formulated with sunflower oil, and fortified with vitamin D, Mg, and Ca checked against two commercially available spreads: No Palm and a well-known commercially available product (RB). The results obtained from the accelerated shelf-life testing for peroxide value (PV) showed relatively higher activation energy (Ea, 14.48 kJ/mol K) for RB, whereas lower Ea (11.31–12.78 kJ/mol K) was obtained for No Palm and all the experimental spread chocolates. Q10 values were comparable (1.202–1.154), indicating a similar catalytic effect of the temperature upon the oxidation rate across all the investigated samples. The positive Gibbs free energies ranged from 75.014 to 83.550 kJ/mol and pointed out that the lipid oxidation reaction in the chocolate spread was an endergonic process. The predicted shelf-life at 293.15 K was 8.57 months (RB), 7 months (No Palm), and 6.8 months for all the experimental spreadable chocolate. However, the higher production of hydroperoxides was observed in chocolate fortified with magnesium-calcium carbonate nanoparticles and stored at 313.15 and 323.15 K, suggesting these particles may enhance lipid oxidation.

## 1. Introduction

Chocolate is generally a high-energy product with a unique taste and texture and contains many carbohydrates and saturated fats [1]. Chocolate spreads are a complex multiphase system of solid-oil suspensions, in which a mix of fats represents the oil phase, whereas sugar, cocoa powder, milled and roasted nuts, dried milk, and whey represent the dispersed phase [2].

Although chocolate spreads are often considered as occasional indulgences and luxury products, the high concentration of saturated fatty acids and sugar limits their use by health-conscious consumers. Saturated fatty acids are harmful to health, especially in terms of cardiovascular diseases, and high sugar consumption is strongly linked to diabetes [3,4,5]. Considering this, chocolate manufacturers are actively working to meet the needs of lowering the amount of unhealthy saturated fats and sugar. However, their reduction could be challenging, as the final product must retain its sensory properties and physical characteristics [6,7]. Since a chocolate spread is a very palatable “comfort food” consumed by the majority of the population, experimental products with low saturated fatty acids and without added sugars seem to be a valuable matrix for the fortification with bioactive health-promoting compounds [5]. Vitamin D is a fat-soluble vitamin primary produced in the human body by the UVB-induced conversion of 7-dehydrocholesterol in the skin. The various stages of vitamin D activations are strongly dependent on the magnesium concentration [8]. Vitamin D acts as a steroid hormone and performs multiple functions in the human body influencing the bones, intestines, immune and cardiovascular systems, pancreas, muscles, brain, and the control of cell cycles [9]. Nowadays, hypovitaminosis D is common worldwide and has become a serious public health concern [10]. Calcium is required by the human body not just to build and maintain strong bones but is also involved in muscles and blood vessels’ contraction, enzymes and hormones secretion, and in sending messages through the nervous system. The calcium requirement depends on age, and growing children and teenagers need more calcium than young adults. A low calcium level increases the risk for osteoporosis in adults and impacts children’s potential height at an adult age [11]. Calcium, along with vitamin D, may have benefits beyond bone health and may protect against cancer, diabetes, and high blood pressure. In our previous research, new spreadable chocolates without palm oil and with no added sugar fortified with vitamin D and Mg and Ca were evaluated in terms of rheology, polyphenols content, in vitro digestion, and sensory acceptability, and compared to well-known commercially available products. The obtained results demonstrated that the incorporation of nanoparticles could affect the rheological, sensorial, and physio-chemical properties of the spreadable chocolates. However, the comparable acceptability of the experimental formulations with the current products on the market was observed [5]. Here, we extend the work by studying the oxidative stability of these formulations which would determine their shelf-life.

On the other hand, palm oil, used in many popular chocolate spreads, is rich in saturated fatty acids known to increase disease risk. Oils rich in unsaturated fatty acids are widely used as a substitute for palm oil. However, they are prone to oxidation with a generation of high amounts of lipid oxidation products such as peroxides, ketones, and aldehydes which can compromise food quality and may result in biological effects with serious health consequences [5,12]. The replacement of nonreducing sugars, such as sucrose, could have negative effects on oxidative stability since sucrose has been shown to inhibit lipid oxidation by altering the food matrix viscosity, changing water activity, scavenging free radicals via its hydroxyl groups, and donating hydrogen [13]. Moreover, the presence of minerals, added in the form of Mg-CaCO_3_ nanoparticles, during storage could promote oxidation [14]. Accelerated shelf-life testing (ASLT) is often used to assess the shelf-life of products, such as cosmetics, pharmaceuticals, and foods. This approach allows a reduction in the time needed to evaluate the shelf-life of a product by accelerating the quality depletion kinetics with increased storage temperature [15]. The approach is widely accepted as the temperature can be easily modified and controlled during the test. Moreover, the temperature dependence of quality depletion can be easily modeled by the Arrhenius equation [16]. Considering what has been reported so far, in this study the peroxidation reaction of no added sugar experimental chocolate spreads formulated without palm oil and fortified with vitamin D, Mg, and Ca with reference to well-known commercially available products was evaluated. In the second stage of the study, the Arrhenius kinetic model was applied to predict the shelf-life, and the thermodynamic behavior of the different chocolate spreads was investigated.

## 2. Materials and Methods

### 2.1. Materials

Standard hydroperoxide, para-anisidine, and all other reagents and solvents used for assessing the oxidation stability were purchased from Sigma-Aldrich (Milan, Italy).

### 2.2. Raw Materials

The major chocolate spread brand (used as a reference brand and so identified as RB) and commercial chocolate cream obtained without palm oil and here coded as “No Palm” were purchased from a local market. All the samples were five months old, according to the production lot (all the spreads were produced in January 2020 and analyzed in June of the same year). The ingredient list of the two spreadable chocolates, as reported on the label, was sugar, palm oil, hazelnuts (13%), skimmed milk powder (8.7%), low-fat cocoa (7.4%), emulsifier: lecithin (soy), vanillin for “RB” cream and sugars, vegetable oils (sunflower and shea oil) hazelnuts (13%), skimmed milk powder, low-fat cocoa powder, whey powder, lactose, emulsifier: soy lecithin, and aroma (vanillin) for “No Palm”. The experimental chocolate spread without palm oil and sugar (NPNS) contained the following ingredients: maltitol, sunflower oil, hazelnuts (13%), skimmed milk powder, whey powder, inulin, low-fat cocoa powder, cocoa butter, lecithin as emulsifier, and vanilla extract, and the relative nutritional values are shown in Table 1. The chocolate spread’s composition was computed based on data provided on the product labels. Inductively coupled plasma mass spectrometry (ICP-MS) has been widely employed for the detection of elements in chocolate [5]. The vitamin D concentration of the samples NPNS, NPNS-VD, NPNS-Ca, and NPNS-VDCa was quantified using an Ultra-Pressure Liquid Chromatography-tandem mass spectrometer (UPLC-MS/MS) with a UPLC Column (Waters Acquity UPLC, HSS T3 2.1 × 100 mm, 1.8 um) as previously reported [5].

### 2.3. Preparation of Magnesium-Calcium Carbonate Nanoparticles

Using a co-precipitation technique, magnesium-calcium carbonate nanoparticles were produced by mixing 0.1 M sodium carbonate solution and 0.1 M calcium chloride containing 0.1 M magnesium chloride for 10 s followed by centrifugation and washed 4 times with ethanol. The obtained particles were dried at 323.15 K in an oven (I. S. Co Srl, Milano, Italy) until a stable weight had been reached.

### 2.4. Preparation of Chocolate Spread Samples

Starting from the NPNS spreadable chocolate, three experimental creams (NPNS-VD, NPNS-Ca, and NPNS-VDCa) were produced. Spreadable chocolates were enriched with a concentration useful to cover 30% of the recommended daily allowance (RDA) for each bioactive compound. NPNS-VD was fortified with 166 mg/kg for vitamin D, NPNS-Ca with 11.6 g/kg of Mg-CaCO_3,_ and NPNS-VDCa with 166 mg/kg for vitamin D and 11.6 g/kg of Mg-CaCO_3_. All the experimental (NPNS-VD, NPNS-Ca, and NPNS-VDCa) and the commercially available chocolate spreads (No Palm and RB) were subjected to the same thermal stress (heating them at 318.15 4 K for 15 min) and mechanical stress, then cooled down to 307.15–309.15 K and stored at 288.15–292.15 K for a week before analysis.

### 2.5. Moisture and Water Activity

The spreadable chocolates’ moisture content was measured on 5 g of the sample using a ventilated oven set at 105 ± 2 °C until a constant weight. The moisture was then calculated as follows (Equation (1)):

(1) $$\mathrm{Moisture}\;(\%)=\frac{\mathrm{weight}\;\mathrm{of}\;\mathrm{fresh}\;\mathrm{sample}\;\left(g\right)-\;\mathrm{weight}\;\mathrm{of}\;\mathrm{dried}\;\mathrm{sample}\;\left(g\right)}{\mathrm{weight}\;\mathrm{of}\;\mathrm{fresh}\;\mathrm{sample}\;\left(g\right)}$$

Water activity (a_w_) was measured using the Hygropalm HC2-AW analyzer (Rotronic Italia Srl, Milano, Italy) at 25 °C.

### 2.6. Storage Condition

Five grams of each formulation (NPNS-VD, NPNS-Ca, NPNS-VDCa, No Palm, and RB) were stored in nine glass tubes covered with aluminum foil and stored at 288.15 K/70% RH, 303.15 K/60% RH, 313.15 K/75% RH, and 323.15 K/75% RH for 31 days. The stability test storage of the formulations was designed based on the International Conference on Harmonization (ICH) climatic zones considering that the products will be distributed and sold in climatic zone I (temperate) and II (subtropical and Mediterranean) and the manufacturer storage temperature of the chocolate spreads after production (288.15 K and 70% RH). The temperature and relative humidity for the intermediate and accelerated stability test storage conditions by ICH for products intended to be stored at room temperature were 30 ± 2 °C/65 ± 5% RH and 40 ± 2 °C/75 ± 5% RH. The accelerated test conditions for the formulations for refrigerated storage were 25 ± 2 °C/60 ± 5% RH [17]. The relative humidity (RH%) was controlled with saturated salt solutions [18]. In detail, a saturated solution of NH_4_NO_3_ was used to maintain the RH of 70 and 60% at 288.15 and 303.15 K, respectively. A saturated NaCl solution was used to maintain the RH of 75% at 313.15 and 323.15 K. At scheduled times (day 0, 1, 3, 5, 8, 12, 16, 20, and 31), one tube was taken from the incubator for hydroperoxides and para-anisidine value determination. The results from the study at this set of temperatures were used to evaluate the oxidative stability, the thermodynamic parameters, and to predict the shelf-life with the Arrhenius equation.

### 2.7. Hydroperoxide Value

Hydroperoxide concentrations were determined from triplicate samples using a method based on the hydroperoxides oxidation of ferrous ions (Fe^2+^) to ferric ions (Fe^3+^), which were then complexed by thiocyanate. Lipid hydroperoxides were extracted by mixing 0.3 g of each chocolate sample with 1.5 mL of isooctane/2-propanol (3:1 *v*/*v*) in a 2 mL Eppendorf for 10 s × 5 times with 3 min between. This was followed by benchtop centrifugation at 778× *g* for 5 min. The upper layer was then filtered, and 0.20 mL of the clear part was collected and mixed with 2.8 mL of methanol/1-butanol (2:1 *v*/*v*), 30 μL of thiocyanate/Fe^2+^ solution. The thiocyanate/ Fe^2+^ solution was prepared in two steps as follows: (1) a solution of 30% of ammonium thiocyanate dissolved in water was prepared; (2) Barium chloride dihydrate was dissolved in 0.4 M HCl to form a 0.8% (*w*/*v*) solution. A given volume of this solution was then mixed with an equal volume of 1% ferrous sulfate heptahydrate dissolved in distilled water followed by centrifugation for 10 min at 3082× *g* a given volume of the clear upper layer, and a solution of Fe (II) Chloride (FeCl_2_) was mixed with an equal volume of solution (1) to form a solution of thiocyanate/Fe^2+^ [19]. The reaction was allowed to take place for 20 min, and the absorbance was measured at 510 nm against a blank that contained all reagents and isooctane/2-propanol instead of the sample, using a spectrophotometer (Scientific Laboratory Supplies Ltd., Nottingham, UK). The concentration of hydroperoxides was calculated from a cumene hydroperoxide standard curve (0–0.4 mM).

### 2.8. Para-Anisidine Value (p-AnV)

The secondary products of oxidation were measured following the AOCS Official Method Cd 18–90 [20]. This method is based on the reactivity of aldehydes with p-anisidine in acetic acid, resulting in a complex that absorbs at 350 nm. One gram of chocolate spread was mixed with 24 mL of isooctane using a vortex mixer followed by centrifugation 20 min at 3082× *g*. Five mL of the top layer was pipetted into glass tubes. One mL of p-anisidine reagent (0.25 g dissolved in 100 mL of glacial acetic acid) was added to each tube, and the sample was mixed for 3 s. After 10 min of incubation, the sample absorbance was measured at 350 nm. The absorbance of the extract without the p-anisidine reagent was also measured. Results were expressed in terms of the p-anisidine value (p-AnV), calculated using Equation (2):

(2) $$\mathrm{p-AnV}=5\;\frac{1.2\;\left(\mathrm{As}-\mathrm{Ab}\right)}{m}$$

where As is the absorbance of the sample before reaction, and Ab is the absorbance of the sample after the reaction with p-anisidine; m is the mass in grams of the sample.

### 2.9. TOTOX Value

The TOTOX value is a useful measure of the initial degradation of fat that provides good information on both primary and secondary products of oxidation. The TOTOX value was calculated according to Equation (3) [21].

TOTOX value = 2PV + p-AnV (3)

### 2.10. Study of Oxidative Stability

The approach for an accelerated shelf-life test (known as ASLT) involves the measurement over time of the selected quality indices, and the application of a mathematical model. For many food products, the loss of quality over time, i.e., the kinetic of variation of a specific quality index, can be modeled, at a constant temperature, using Equation (4):

(4) $$\frac{\mathrm{dA}}{\mathrm{dt}}={\mathrm{kA}}^{n}$$

where k is the specific kinetic constant, that is the reaction rate constant; t is the time; A is the measurable quantity that expresses the quality index, which is PV, p-AnV, or TOTOX in the current study; and n is the power factor called the reaction order. The order of reaction has been estimated for zero, first, and second-order reactions. The regression equation has thus been obtained by plotting the value of A in the natural logarithmic form or the value of 1/A as a function of time for the estimation of the zero, first, and second-order equations. After this step, the R^2^ of the regression curve was compared and the best fit model was chosen for the kinetic and thermodynamic studies. Almost all food quality decay reactions are strongly influenced by temperature, and the dependence of their kinetics on system temperature can be effectively described by Arrhenius’ law Equation (5):

(5) $$k={\;k}_{0}\times {\;e}^{\frac{-E_{a}}{\mathrm{RT}}}$$

where k is the specific reaction kinetic constant, k_0_ is the pre-exponential factor, E_a_ is the activation energy (kJ/mol K) that is the minimum energy necessary to trigger the reaction, R is the gas universal constant (8.318 kJ/mol K), and T is the absolute temperature (K).

By collecting the experimental data relating to the reaction rates of a product and knowing the relationship that binds them to the chosen acceleration factor, it is possible to estimate the rate of decay or the shelf-life in the conditions of interest in a real commercial setting or industrial production. By plotting the natural logarithm of the reaction rate versus the inverse absolute temperature for each index, several attributes describing the kinetics of indices, namely oxidation reaction rate and activation energy of the reaction, could be estimated from the Arrhenius equation [22]. Moreover, it was possible to estimate the shelf-life by using the obtained k value in Equation (6):

(6) $$t=\frac{{\mathrm{lnA}}_{t}-{\mathrm{lnA}}_{0}}{k}.\;$$

where A_0_ is the quality index value at the beginning of the shelf-life, A_t_ is the limit value of the quality index, k is the specific kinetic constant, and t is the shelf-life. The oxidative stability of the chocolate spread was considered as the time required for the cream’s peroxide value to reach 10 m_eq_ O_2_/kg, which could be considered as the beginning of the alteration of the fat phase of the product [23,24]. In addition, it can be useful to calculate Q_10_ using Equation (7), representing the change in the rate of oxidation products’ formation with a 10 K change in temperature [25]:

(7) $$Q_{10}={\;e}^{\frac{10E_{a}}{\mathrm{RT}\left(T\;+10\right)}}\;$$

The Q_10_ was calculated considering the temperatures of 303.15 and 313.15 K.

#### Thermodynamics of Lipid Oxidation

The constant rate “k” and activation energy (E_a_) were used to determine the Gibbs free energy change (G), the enthalpy change (ΔH), and entropy change (ΔS), according to Equations (8)–(10) as reported by Lago and Noreña [26].

(8) $$\Delta G=-\mathrm{RT}\ln\frac{\left({\mathrm{kh}}_{p}\right)}{\left(K_{b}T\right)}.\;$$

(9) $$\Delta H={\;E}_{a}-\mathrm{RT}$$

(10) $$\Delta S=\frac{\Delta H-\Delta G}{T}$$

where h_p_ and K_b_ are the Planck (6.6262 × 10^−34^ J s) and Boltzmann (1.3806 × 10^−23^ J/ K) constants, respectively.

### 2.11. Statistical Analysis

The analyses were carried out in triplicates and the mean values ± standard deviation were reported. The variables were tested for significance using a one-way analysis of variance (ANOVA). Differences among means were assessed using Tukey’s HSD tests (*p* < 0.05). All the statistical analyses were carried out using XLSTAT software (XLSTAT Premium Version 2019.4.2, Addinsoft, Paris, France).

## 3. Results

### 3.1. Oxidative Stability of Spreadable Chocolates

**Figure 1:**
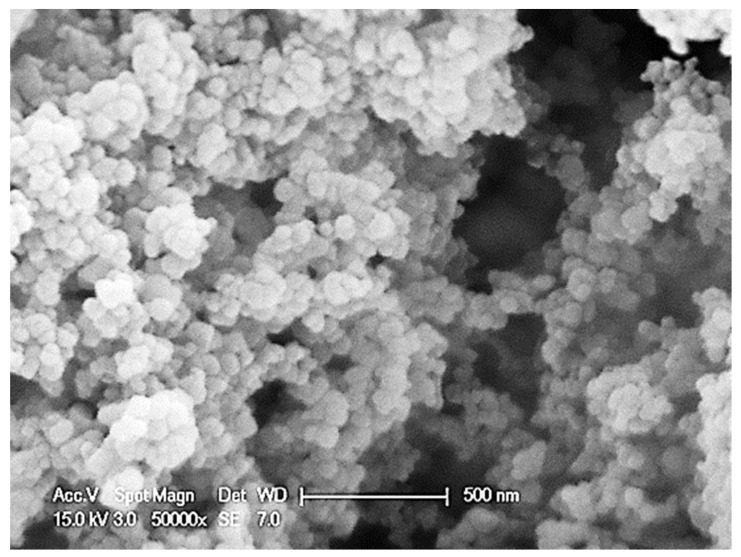
Scanning electron microscope image of magnesium-calcium carbonate nanoparticles (Mg-CaCO_3_) at magnification 50,000.

The oxidation stability of spreadable chocolates fortified with vitamin D_3_ and Mg and calcium using Mg-CaCO_3_ nanoparticles was evaluated by measuring the concentration of primary and secondary oxidation products, and TOTOX values after the samples’ incubation at four different test temperatures for 31 days. As is possible to observe from the scanning electron microscope image reported in Figure 1, the Mg-CaCO_3_ nanoparticles were prone to agglomeration. However, these agglomerated particles exhibited a small particle size [5].

**Figure 2:**
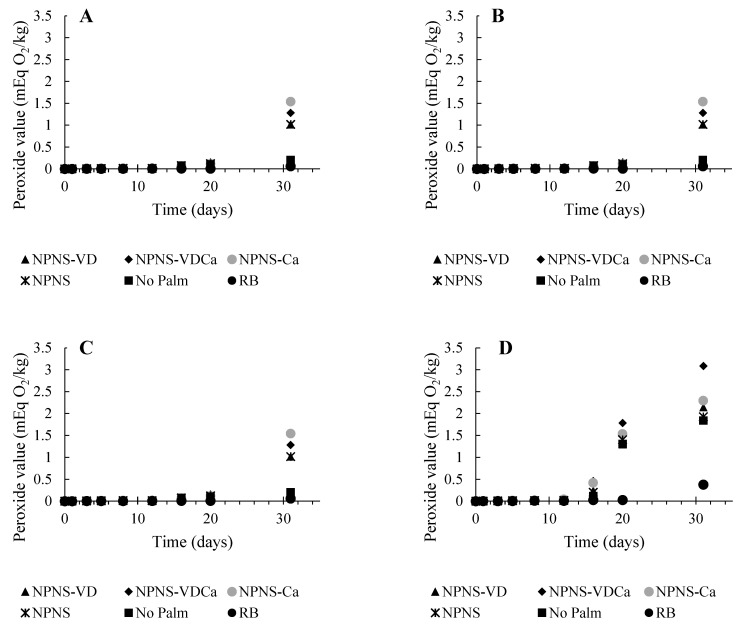
Peroxide value (mEq O2/kg) of the fat assessed for different chocolate spreads over 31 days of storage at 288.15 K (**A**), 303.15 K (**B**), 313.15 K (**C**), and 323.15 K (**D**). RB, reference brand; NPNS, spreadable chocolate without palm oil and sugar; NPNS-VD, spreadable chocolate without palm oil and sugar fortified with vitamin D; NPNS-VDCa, spreadable chocolate without palm oil and sugar fortified with vitamin D and Mg-CaCO_3_ nanoparticles; NPNS-Ca, spreadable chocolate without palm oil and sugar fortified with Mg-CaCO_3_ nanoparticles.

The oxidation rate of the oil extracted from the chocolate spreads kept in the accelerated storage conditions was determined by plotting the PV, p-AnV, and TOTOX values versus storage time (days). Since the hydroperoxide compounds are the primary products of lipid oxidation, measuring the formation of these products is considered a useful index for monitoring the oxidation reactions [27]. The official methods proposed by the AOAC [28] and the AOCS [29] are both based on an iodometric titration for the evaluation of the PV and have low sensitivity while requiring a large amount of lipid samples. Thus, to improve the drawbacks of the official methods, several new methods were developed. In this study, a protocol based on the Official International Dairy Federation as reported by Zhang et al. [30] has been applied. This spectrophotometric method is highly sensitive and requires a small amount of sample (<0.02 g). In Figure 2, the PVs of the spreadable chocolates stored at 288.15, 303.15, 313.15, and 323.15 K plotted versus time are reported.

**Figure 3:**
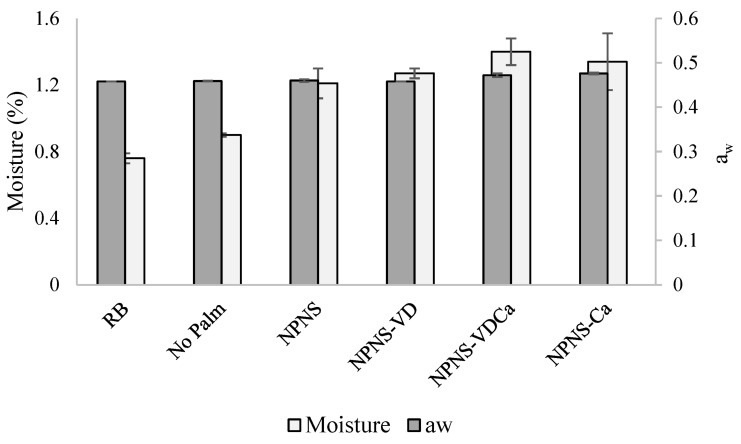
Moisture (%) and water activity (aw) of the spreadable chocolates. RB, reference brand; NPNS, spreadable chocolate without palm oil and sugar; NPNS-VD, spreadable chocolate without palm oil and sugar fortified with vitamin D; NPNS-VDCa, spreadable chocolate without palm oil and sugar fortified with vitamin D and Mg-CaCO_3_ nanoparticles; NPNS-Ca, spreadable chocolate without palm oil and sugar fortified with Mg-CaCO_3_ nanoparticles.

The levels of PV at the zero time of storage were comparable among the different spreadable chocolates. The values ranged between 4.9 × 10^−4^ for the RB chocolate spread to 9.0 × 10^−4^ meq O_2_/kg oil for NPNS, and there was no significant difference among them. The PV steadily increased during the storage period as highlighted in Figure 2, showing the PV of the spreadable chocolates stored at 288.15, 303.15, 313.15, and 323.15 K plotted versus time. This trend, although observed in all formulations, was less marked for the RB sample. These results are consistent with the RB composition, characterized by high saturated fat concentration, and could also be linked to the lower water activity (a_w_) and moisture content of the RB formulation (Figure 3).

**Figure 4:**
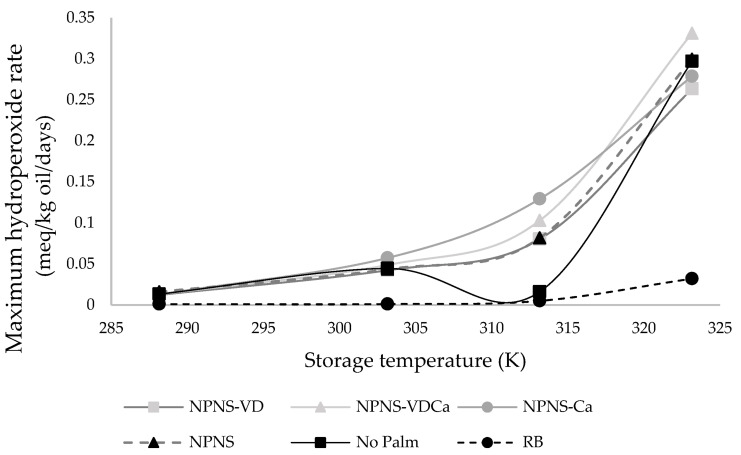
Maximum rate hydroperoxides production plotted as a function of the storage temperature. RB, reference brand; NPNS, spreadable chocolate without palm oil and sugar; NPNS-VD, spreadable chocolate without palm oil and sugar fortified with vitamin D; NPNS-VDCa, spreadable chocolate without palm oil and sugar fortified with vitamin D and Mg-CaCO3 nanoparticles; NPNS-Ca, spreadable chocolate without palm oil and sugar fortified with Mg-CaCO3 nanoparticles.

As reported by Vu et al. [31], in low moisture food, a_w_ is one of the factors that could impact lipid oxidation. Moreover, the scrutiny of data reported in Figure 2 and Figure 3 revealed a reduction in the lag phase for the PV with an increased moisture content of the spreadable creams. Figure 4 represents the plot of the maximum rate of hydroperoxide production as a function of the temperature storage.

As a general rule, the maximum amount of hydroperoxides produced increased slowly and almost linearly from 288.15 K to 303.15 K before starting to sharply rise according to the storage temperature. A pronounced increase rate was found in NPNS samples, where the chocolate spread without palm oil and sugar fortified with vitamin D and Mg-CaCO_3_ nanoparticles (NPNS-VDCa) showed an increased rate of 0.33 meq O_2_/kg oil per day, followed by the based-formulation chocolate spread without palm oil and sugar (NPNS) and No Palm formulation. As expected, the RB sample showed the lowest hydroperoxides rate with a 10-fold lower value than the NPNS-VDCa sample (0.032 meq O_2_/kg oil per day). The results also showed the existence of a negative inflexion for No Palm chocolate spread at 313.15 K, which could be attributed to the change in the food microstructure above the melting point of cocoa butter that is around 306.15 K [32], increasing pro-oxidants and antioxidant mobility. The catalytic effect of the temperature on the oxidation, associated with the increased reactants’ mobility, could explain the observed sharp increase in PV at higher temperatures. The peroxide value provides a measure of the products formed at the early stages of oxidation and normally shows a high value during the primary stages of oxidation followed by a decline due to the decomposition of the hydroperoxides into secondary products such as aldehydes and ketones. These secondary products are then estimated via the para-anisidine value (p-AnV) [22,33]. There was no significant change in the p-AnV over the 31 days storage period for all the samples (Figure S1), indicating no apparent degradation of the hydroperoxide compounds throughout the investigated storage times. The initial value of p-AnV was high for the samples available in the market (No Palm > RB) when compared to the experimental samples; however, the increase in the storage temperature from 288.15 to 323.15 K did not lead to a significant increase in p-AnV. The delay in secondary oxidation product formation, as expressed by the relatively stable values of p-AnV over the studied period when compared to PV, could be explained by the fact that low moisture foods have a high amount of bound water and low molecular mobility. For example, the low mobility of metals in high viscous and low water content products would restrain their catalytic activity and ability to induce the decompose hydroperoxide compounds into secondary oxidation products [34]. Similarly, Vu et al. [31] hypothesized that the delay in the secondary oxidation product could be ascribed to water binding hydroperoxide compounds, thereby inhibiting their decomposition. Or, as hypothesized by Yang and Boyle [35], this trend could be due to the altered oxidation pathways or reaction rates of lipids under different storage conditions. These observations made the latter authors conclude that although p-AnV measures the secondary oxidation products, its low specificity and tendency to be influenced by other factors, such as the physical states of lipids, fatty acid compositions, and storage conditions, greatly limit its application. The TOTOX value of the samples followed the same trend as p-AnV, since it was expressed by 2PV + p-AnV and the value of PV was relatively smaller when compared to p-AnV (Figure S2). Based on these results of p-AnV and TOTOX, only data from the PV analysis were used to assess the oxidative stability and shelf-life estimation of all the samples.

### 3.2. Kinetic Study of Spreadable Chocolates Oxidation

The production of hydroperoxide compounds is generally described both by the zero and first-orders’ kinetic reaction [22,36]. In the present study, the oxidation kinetics were best described by the first-order model with the highest correlation coefficients (Table 2), though the RB sample at 303.15 K showed a better fit by the zero-order kinetic with R^2^ = 0.957 when compared to 0.662 for the second-order model. Indeed, the first-order model showed R^2^ between 0.8214 and 0.9465. The first-order kinetics have been previously applied by different authors to describe hydroperoxide compounds’ production and changes during food storage [36,37]. Our results are in agreement with the study by Hosseini et al. [37], which reported that the oxidation reaction in walnut kernels followed the first-order kinetic. On the other hand, the second reaction order regression showed the smallest value of R^2^ especially at higher temperatures, 313.15–323.15 K (R^2^ generally below 0.3). The first-order equation was therefore chosen for the estimation of k, E_a_, thermodynamic parameters, and product shelf-life.

Table 3 shows the correlation equations describing the formation of hydroperoxides, the R^2^, and the kinetic constant of hydroperoxides production at different temperatures for the experimental and commercially available spreadable chocolates.

As expected, the peroxide value showed a significant increase at elevated temperatures. Indeed, the kinetic constant k_PV_, that is the angular coefficient of the regression equation obtained by plotting PV vs. time, increased with the increased temperature. The maximum k_PV_ was observed for NPNS-VDCa at 323.15 K, and the lowest k_PV_ was recorded for the RB sample at 288.15, 303.15, and 313.15 K.

### 3.3. Shelf-Life Estimation of Chocolate Spreads

**Figure 5:**
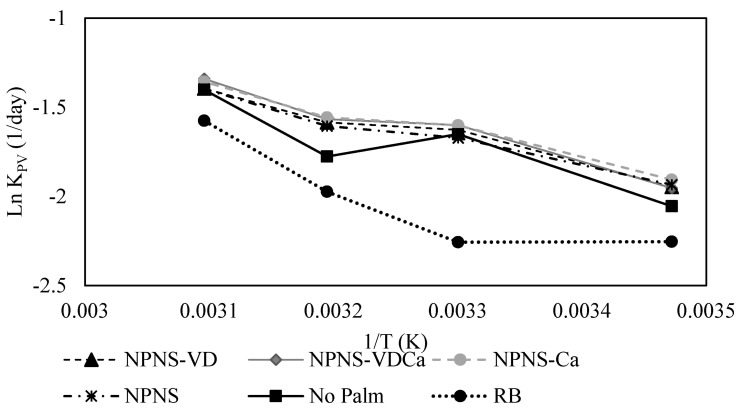
Arrhenius graph from plotting the natural logarithm of the rate constant Ln k_PV_ for chocolate spread stored at 288.15, 303.15, 313.15, and 323.15 K against reverse absolute temperature (1/K). RB, reference brand; NPNS, spreadable chocolate without palm oil and sugar; NPNS-VD, spreadable chocolate without palm oil and sugar fortified with vitamin D; NPNS-VDCa, spreadable chocolate without palm oil and sugar fortified with vitamin D and Mg-CaCO_3_ nanoparticles; NPNS-Ca, spreadable chocolate without palm oil and sugar fortified with Mg-CaCO_3_ nanoparticles.

Because of the pressure to maintain the market share with new and innovative products development and launching, it is common in the food industry to use accelerated shelf-life tests (ASLT), especially for ambient stable products with a long shelf-life (≥6 months). In this study, the Arrhenius plots, obtained by the natural logarithm of the rate constant Ln k_PV_ for chocolate spreads stored at 288.15, 303.15, 313.15, and 313.15 K against the inverse absolute temperature (1/K) (Figure 5), was performed as a means of a predictive calculation of the product shelf-life’s common storage temperature (293.15 and 298.15 K).

As explained before, according to the coefficients of linear regression (R^2^), the kinetic model that fitted the results from the oxidation of spreadable chocolate, assessed both in terms of PV, p-AnV, and TOTOX, corresponded to a first-order model equation for all the formulations (Table 2). From the Arrhenius plot of the degradation index, PV selected based on the R^2^, it was possible to calculate the Arrhenius shelf-life equations, the activation energy (Ea), as well as the Q10 factor for hydroperoxides production (Table 4).

The different formulations were characterized by different Ea values. Generally, the Ea for the lipid oxidation of the spreadable chocolates ranged from 11.31 to 14.48 kJ/mol K, which was under the lowest range estimated for lipids rancidity reactions (24–240 kJ/mol K) [38,39]. The Ea of the RB sample was higher than that of other formulations, indicating, as expected, its higher oxidative stability followed by the NPNS-VDCa and No Palm samples. The Q10 values ranged from 1.202 to 1.154, indicating a similar catalytic effect of the temperature upon the oxidation rate across all the investigated chocolate spreads. The Q10 coefficient represents the increase in the rate of a process with an increase of 10 K of the storage temperature. In general, the rate of chemical processes doubles or triples when the temperature is raised by 10 °C. The values obtained in the accelerated experiment were used to calculate the shelf-life of the different chocolate spreads a function of the relative increase in the PV using the starting hydroperoxide value, the k_PV_, and the Ea values. Table 4 shows that the shelf-life of all the chocolate spread creams was obviously strongly temperature-dependent with a longer shelf-life obtained at 293.15 K when compared to 298.15 K.

According to our results, sample RB showed the longer shelf-life followed by the No Palm, and finally the experimental samples (NPNS, NPNS-VD, NPNS-VDCa, and NPNS-Ca). This is an expected result since palm oil, rich in saturated fatty acids, was replaced in the experimental chocolate samples by high oleic sunflower oil which contains a significant concentration of unsaturated fatty acids including polyunsaturated fats [40]. As mentioned above, oils rich in unsaturated fatty acids and the resulting products are prone to oxidation [41].

It was apparent that fortification, and especially the addition of magnesium-calcium carbonate nanoparticles in the formulation resulted in a further reduction in the oxidation stability of the formulated samples and products’ shelf-life. Acknowledging the complexity of the lipid oxidation reaction with different interactions between substrates and catalysts in the food matrix where different food components can promote or inhibit the oxidative reactions, the overall oxidative stability would depend on the balance of anti- and pro-oxidant compounds. Moreover, it has been demonstrated that different calcium salts could act as pro-oxidants or antioxidants with calcium ascorbate inhibiting lipid oxidation, whereas calcium lactate and calcium chloride exhibit pro-oxidant properties on treated beef [42]. This raises questions worth investigating about the right calcium source to be used for fortifying chocolate spread and whether coating the nanoparticles in a protective shell or encapsulating vitamin D could improve the oxidation stability of the fortified chocolate creams.

Considering that all the samples were 5 months old at the start of the experiment, the overall estimated shelf-life was 8.57 months (RB), 7 months (No Palm), and 6.8 months for all the experimental creams. It should be emphasized that the shelf-life estimation is just an approximation since, usually, the storage method and the packaging used are different from those used in this experimental evaluation. However, despite the limitations of the ASLT, information obtained on the rate of production of PV and the resulting predicted shelf-life provide a useful comparative insight into the oxidative stability of the developed products in comparison to that of commercially available spreadable chocolates.

### 3.4. Thermodynamic Study of the Chocolate Spreads

Table 5 represents the thermodynamic parameters for all chocolate spread samples. The Gibbs free energies ranged from 75.014 to 83.550 kJ/mol and were positive, which underlined the unspontaneous and endergonic nature of the lipid oxidation reaction in spreadable chocolate. Elhussein et al. [43] showed similar results in their thermodynamic studies of sesame seed oil. The enthalpy ranged from 8.627 to 12.089. The enthalpy values, were positives, and, thus the oxidation reactions, were endothermic. The endothermic nature of the lipid oxidation system was also reported by Elhussein et al. [43] and Tan et al. [44]. The calculated entropy ranged between −230.244 and 222.901 J/K mol, and the lowest value was assessed for the RB chocolate spread, indicating fewer numbers of species in the activated complex state and thus the lipid oxidation being less probable [44]. The negative value of entropy represents a further confirmation of the unspontaneity of the oxidation reaction within the tested system (Table 5).

## 4. Conclusions

A comparative study applying the Arrhenius model was carried out to investigate the oxidation kinetics; thermodynamic parameters; and to predict the shelf-life of newly developed no added sugar chocolate spread creams formulated with sunflower and shea oil, and fortified with vitamin D, Mg, and Ca, using two commercially available spreads, No Palm and a well-known commercially available product (RB) as a reference. The oxidation kinetics were best described by the first-order model with R^2^ between 0.8214 and 0.9465 using the hydroperoxide value (PV) as the lipid oxidation index for samples at different storage temperatures for 31 days. Thermodynamic parameters suggested that the lipid oxidation reaction in the tested spreadable chocolates was a nonspontaneous endergonic process. The obtained predicted shelf-life was lower for the experimental samples when compared to the commercially available ones: No Palm and the reference brand (RB). While acknowledging the limitations of the accelerating shelf-life testing, especially as the chocolate spreads changed phase at a higher temperature (above 303.15 K) which also altered the mobility of the reactant in the food matrix, thus the oxidation rate, the information obtained on the rate of production of PV and the resulting predicted shelf-life provides a useful comparative insight into the oxidative stability of the developed products against that of the commercially available spreadable chocolates. On the other hand, it was apparent that magnesium-calcium carbonate nanoparticles containing samples generated the highest amount of hydroperoxides, indicating that these nanoparticles could be acting as pro-oxidants. Future work will focus on strategies to enhance the oxidative stability of these experimental chocolate spreads, including the study of the effect of different calcium sources and whether coating the nanoparticles in a protective shell could improve the oxidation stability of the fortified chocolate creams.

## Figures and Tables

**Table 1:** Composition of 100 g of formulated chocolate spreads compared with commercially available one.

KERRYPNX	RB	No Palm	NPNS	NPNS-VD	NPNS-VDCa	NPNS-Ca
Energy (KJ/100 g)	2252	2223	2074	2074	2074	2074
Total fat (g/100 g)	30.90	30.00	36.00	36.00	36.00	36.00
Saturated (g/100 g)	10.60	5.20	6.50	6.50	6.50	6.50
Total carbohydrate (g/100 g)	57.50	58.00	45.00	45.00	45.00	45.00
Sugars (g)	56.30	56.00	13.00	13.00	13.00	13.00
Polyols (g)	-	-	30.00	30.00	30.00	30.00
Fiber (g)	-	-	9.80	9.80	9.80	9.80
Protein (g)	6.30	6.10	6.40	6.40	6.40	6.40
NaCl salt (g)	0.11	0.08	0.14	0.14	0.14	0.14
Vitamin D (μg/100 g)	-	-	<0.50	15.20	15.10	<0.50
Calcium	1.65	1.36	2.28	2.61	10.22	10.64
Magnesium	0.84	0.65	0.79	0.84	2.06	2.12

RB, reference brand; NPNS, spreadable chocolate without palm oil and sugar; NPNS- VD, spreadable chocolate without palm oil and sugar fortified with vitamin D; NPNS- VDCa, spreadable chocolate without palm oil and sugar fortified with vitamin D and Mg-CaCO_3_ nanoparticles; NPNS-Ca, spreadable chocolate without palm oil and sugar fortified with Mg-CaCO_3_ nanoparticles.

**Table 2:** Coefficients of correlation R2 for peroxide value of chocolate spread for zero, first, and second-order kinetic equation.

Chocolate Cream Sample	Storage Temperature (K)	R^2^		
		Zero Order	First Order	Second Order
NPNS-VD	288.15	0.61	0.94	0.63
	303.15	0.71	0.86	0.43
	313.15	0.67	0.95	0.32
	323.15	0.81	0.90	0.28
NPNS-VDCa	288.15	0.61	0.93	0.58
	303.15	0.71	0.86	0.43
	313.15	0.65	0.95	0.30
	323.15	0.82	0.89	0.27
NPNS-Ca	288.15	0.62	0.94	0.50
	303.15	0.69	0.86	0.32
	313.15	0.62	0.93	0.26
	323.15	0.83	0.90	0.25
NPNS	288.15	0.60	0.92	0.64
	303.15	0.71	0.87	0.38
	313.15	0.66	0.95	0.33
	323.15	0.79	0.91	0.32
No Palm	288.15	0.59	0.82	0.51
	303.15	0.71	0.89	0.37
	313.15	0.90	0.89	0.37
	323.15	0.77	0.91	0.33
RB	288.15	0.75	0.72	0.43
	303.15	0.96	0.66	0.32
	313.15	0.68	0.72	0.24
	323.15	0.64	0.88	0.24

RB, reference brand; NPNS, spreadable chocolate without palm oil and sugar; NPNS-VD, spreadable chocolate without palm oil and sugar fortified with vitamin D; NPNS-VDCa, spreadable chocolate without palm oil and sugar fortified with vitamin D and Mg-CaCO3 nanoparticles; NPNS-Ca, spreadable chocolate without palm oil and sugar fortified with Mg-CaCO3 nanoparticles.

**Table 3:** Regression equations from variations in oxidative indices during accelerated storage of chocolate spreads.

Storage Temperature	Chocolate Cream	Linear Regression Equation	k _PV_ (1/Day)	Temperature Effect on k _PV_	Formulation Effect on k _PV_	R^2^
**288.15 K**	**NPNS-VD**	y = 0.1428x − 6.9104	0.1428	0.1351	0.1730	0.94
	**NPNS-VDCa**	y = 0.1418x − 6.845	0.1418		0.2026	0.93
	**NPNS-Ca**	y = 0.1487x − 6.8993	0.1487		0.2042	0.94
	**NPNS**	y = 0.1445x − 6.8506	0.1445		0.1952	0.92
	**No Palm**	y = 0.1281x − 6.7932	0.1281		0.1838	0.82
	**RB**	y = 0.105x − 7.8284	0.105		0.1166	0.72
**303.15 K**	**NPNS-VD**	y = 0.1966x − 6.1561	0.1966	0.1808	0.1730	0.86
	**NPNS-VDCa**	y = 0.2019x − 6.1671	0.2019		0.2026	0.86
	**NPNS-Ca**	y = 0.2016x − 6.0942	0.2016		0.2042	0.86
	**NPNS**	y = 0.1879x − 5.9261	0.1879		0.1952	0.87
	**No Palm**	y = 0.1919x − 6.0538	0.1919		0.1838	0.89
	**RB**	y = 0.1047x − 7.3513	0.1047		0.1166	0.66
**313.15 K**	**NPNS-VD**	y = 0.205x − 6.1991	0.2050	0.1832	0.1730	0.95
	**NPNS-VDCa**	y = 0.2087x − 6.1843	0.2087		0.2026	0.95
	**NPNS-Ca**	y = 0.2107x − 6.1757	0.2107		0.2042	0.93
	**NPNS**	y = 0.2009x − 6.1026	0.2009		0.1952	0.95
	**No Palm**	y = 0.1693x − 6.0933	0.1693		0.1838	0.89
	**RB**	y = 0.1047x − 7.3513	0.1047		0.1166	0.72
**323.15 K**	**NPNS-VD**	y = 0.2479x − 6.0102	0.2479	0.2446	0.1730	0.90
	**NPNS-VDCa**	y = 0.2618x − 5.9565	0.2618		0.2026	0.89
	**NPNS-Ca**	y = 0.2577x − 5.9401	0.2577		0.2042	0.90
	**NPNS**	y = 0.2474x − 6.0628	0.2474		0.1952	0.91
	**No Palm**	y = 0.2461x − 6.1799	0.2461		0.1838	0.91
	**RB**	y = 0.2070x − 7.176	0.2070		0.1166	0.88

RB, reference brand; NPNS, spreadable chocolate without palm oil and sugar; NPNS-VD, spreadable chocolate without palm oil and sugar fortified with vitamin D; NPNS-VDCa, spreadable chocolate without palm oil and sugar fortified with vitamin D and Mg-CaCO_3_ nanoparticles; NPNS-Ca, spreadable chocolate without palm oil and sugar fortified with Mg-CaCO_3_ nanoparticles.

**Table 4:** Arrhenius equations, activation energy (E_a_ KJ/mol K), rate constant at 293.15 and 298.15 K (k _293.15_ and k_298.15_), Q10, correlation coefficient value (r^2^), and shelf-life at 293.15 and 298.15 K estimated for different chocolate spread samples.

Chocolate Cream	Arrhenius Equation	E_a_ (KJ/mol K)	k_293.15_ (1/Day) at 293.15 K	k_298.15_ (1/Day) at 298.15 K	Q_10_	R^2^	Estimated Shelf-Life at 293.15 K (Days)	Estimated Shelf-Life at 298.15 K (Days)
NPNS-VD	y = −1394.8x + 2.9172	11.5963	0.1583	0.1715	1.1584	0.96	59.5	55.0
NPNS-VDCa	y = −1537.3x + 3.4057	12.7811	0.1586	0.1732	1.1760	0.95	59.4	54.4
NPNS-Ca	y = −1386.0x + 2.9212	11.5232	0.1638	0.1773	1.1574	0.96	59.1	54.6
NPNS	y = −1360.6x + 2.7915	11.3120	0.1569	0.1696	1.1543	0.97	59.4	54.9
No Palm	y = −1503.4x + 3.189	12.4992	0.1434	0.1563	1.1718	0.79	65.6	60.2
RB	y = −1742.1x + 3.6748	14.4838	0.1032	0.1140	1.2016	0.76	107.3	97.1

RB, reference brand; NPNS, spreadable chocolate without palm oil and sugar; NPNS-VD, spreadable chocolate without palm oil and sugar fortified with vitamin D; NPNS-VDCa, spreadable chocolate without palm oil and sugar fortified with vitamin D and Mg-CaCO_3_ nanoparticles; NPNS-Ca, spreadable chocolate without palm oil and sugar fortified with Mg-CaCO_3_ nanoparticles.

**Table 5:** ΔG, ΔH, and ΔS of chocolate spread formulations. ΔG and ΔH are expressed as kJ/mol. Only mean value of ΔS (at different temperatures) is reported.

Chocolate Spread	ΔG (kJ/mol)				ΔH (kJ/mol)				ΔS (J/K mol)
	288.15 K	303.15 K	313.15 K	323.15 K	288.15 K	303.15 K	313.15 K	323.15 K	Mean Value
NPNS-VD	75.111	78.346	80.907	83.066	9.202	9.077	8.994	8.911	−229.200 + 0.555
NPNS-VDCa	75.128	78.279	80.861	82.920	10.387	10.262	10.179	10.096	−225.139 + 0.612
NPNS-Ca	75.014	78.283	80.836	82.962	9.129	9.004	8.921	8.838	−229.165 + 0.544
NPNS	75.083	78.460	80.960	83.072	8.918	8.793	8.710	8.627	−230.244 + 0.502
No Palm	75.372	78.407	81.405	83.086	10.105	9.980	9.897	9.814	−226.940 + 1.103
RB	75.848	79.933	81.918	83.550	12.089	11.965	11.882	11.798	−222.901 + 1.369

RB, reference brand; NPNS, spreadable chocolate without palm oil and sugar; NPNS- VD, spreadable chocolate without palm oil and sugar fortified with vitamin D; NPNS- VDCa, spreadable chocolate without palm oil and sugar fortified with vitamin D and Mg-CaCO_3_ nanoparticles; NPNS-Ca, spreadable chocolate without palm oil and sugar fortified with Mg-CaCO_3_ nanoparticles.

## Data Availability

Data is contained within the article or supplementary material.

## Supplementary Materials

The following supporting information can be downloaded at: https://www.mdpi.com/article/10.3390/foods11152358/s1, Figure S1 Para-anisidine of the fat extracted from different chocolate spreads over 31 days of storage at 288.15 K (A), 303.15 K (B), 313.15 K(C) and 323.15 K (D); Figure S2 TOTOX of the fat extracted from different chocolate spreads over 31 days of storage at 288.15 K (A), 303.15 K (B), 313.15 K(C) and 323.15 K (D).
