# Supplementary material for: Shelf-Life Prediction and Thermodynamic Properties of No Added Sugar Chocolate Spread Fortified with Multiple Micronutrients
Source: Foods. 2022 Aug 6;11(15):2358. doi: 10.3390/foods11152358 (PMC9368434; doi:10.3390/foods11152358)
Supplement: Supplementary file 1 [file foods-11-02358-s001.zip › foods-1828723-Supplementary.pdf]

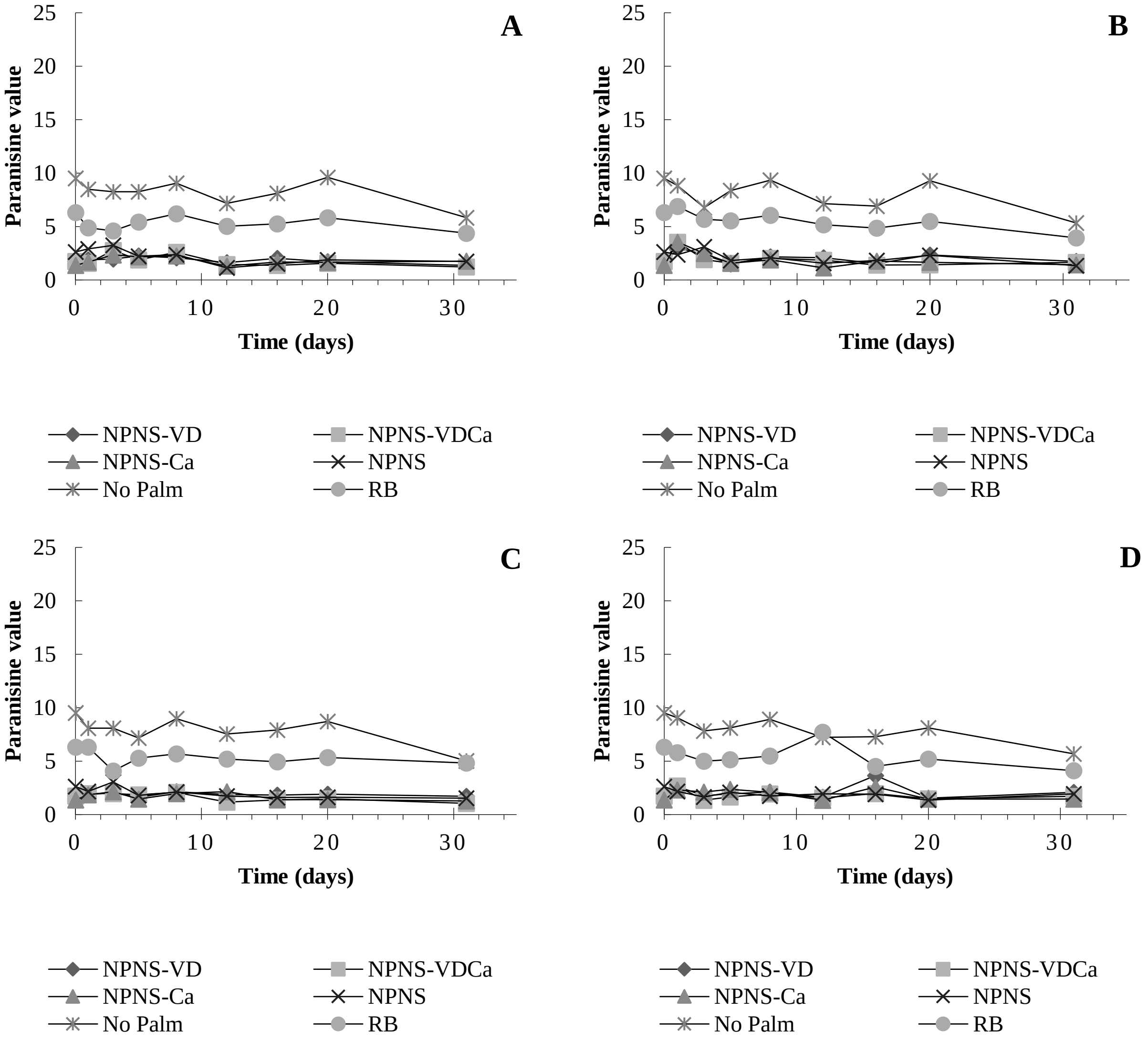

Figure S1. Para-anisidine of the fat extracted from different chocolate spreads over 31 days of storage at 288.15 K (**A**), 303.15 K (**B**), 313.15 K(**C**) and 323.15 K (**D**).

RB Reference brand; NPNS No palm no sugar spreadable chocolate; NPNS- VD No palm no sugar chocolate spreadfortified with vitamin D; NPNS- VDCa No palm no sugar chocolate spreadfortified with vitamin D and Mg-$CaCO_3$ nanoparticles; NPNS-Ca No palm no sugar chocolate spreadfortified with Mg-$CaCO_3$ nanoparticles.

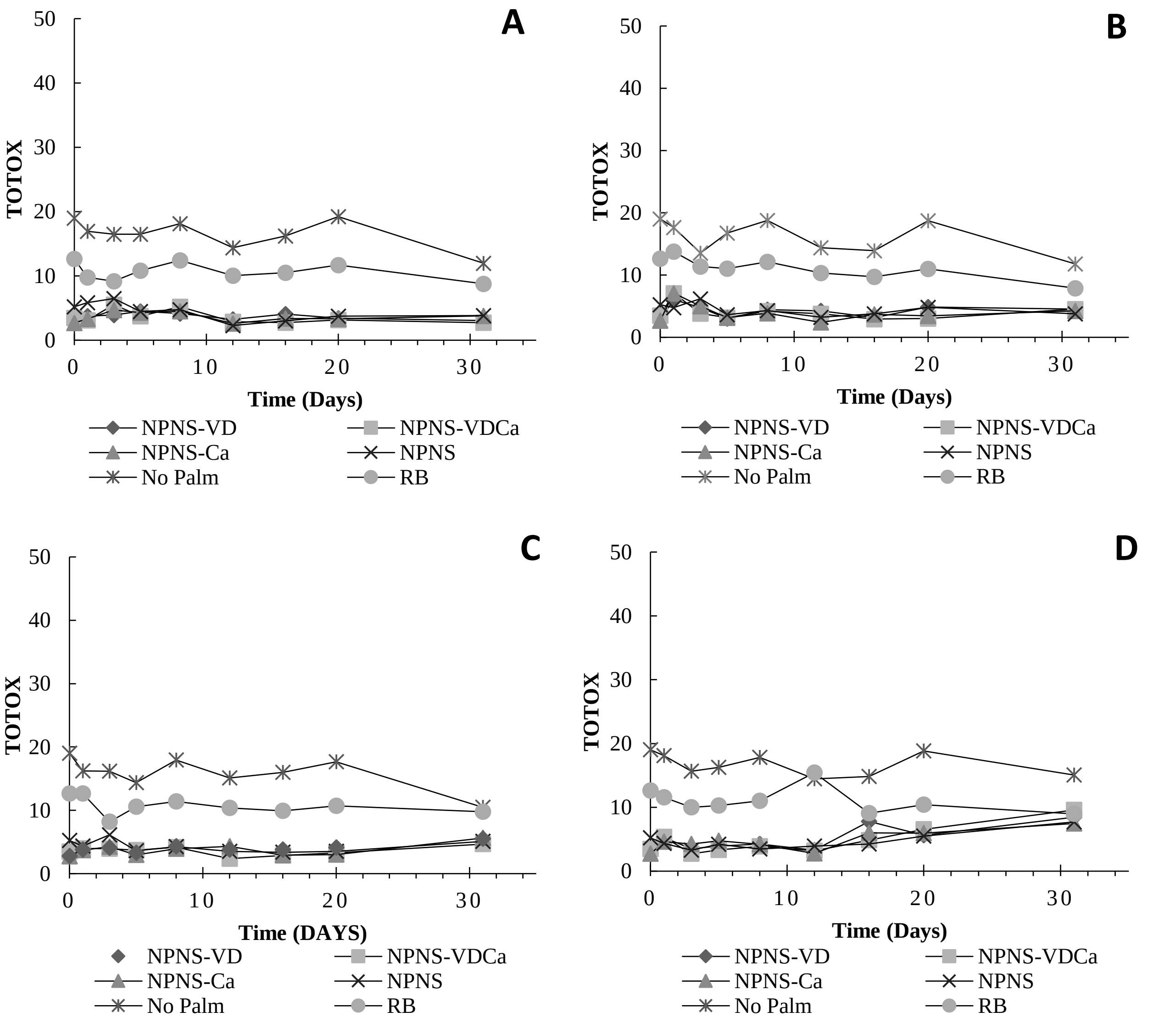

Figure S2 .TOTOX of the fat extracted from different chocolate spreads over 31 days of storage at 288.15 K (**A**), 303.15 K (**B**), 313.15 K(**C**) and 323.15 K (**D**).

RB Reference brand; NPNS No palm no sugar spreadable chocolate; NPNS- VD No palm no sugar chocolate spreadfortified with vitamin D; NPNS- VDCa No palm no sugar chocolate spreadfortified with vitamin D and Mg-$CaCO_3$ nanoparticles; NPNS-Ca No palm no sugar chocolate spreadfortified with Mg-$CaCO_3$ nanoparticles.
